# Raptor and rictor expression in patients with human papillomavirus-related oropharyngeal squamous cell carcinoma

**DOI:** 10.1186/s12885-021-07794-9

**Published:** 2021-01-22

**Authors:** Shunsuke Kondo, Hitoshi Hirakawa, Taro Ikegami, Takayuki Uehara, Shinya Agena, Jin Uezato, Hidetoshi Kinjyo, Noritomo Kise, Yukashi Yamashita, Katsunori Tanaka, Narumi Hasegawa, Asanori Kiyuna, Hiroyuki Maeda, Mikio Suzuki, Akira Gahana

**Affiliations:** 1 Department of Otorhinolaryngology, Head and Neck Surgery, Graduate School of Medicine, University of the Ryukyus, 207 Uehara, Nishihara-cho, Okinawa, 903-0215 Japan; 2 Ryukyu Society for the Promotion of Oto-Rhino-Laryngology, Nishihara-cho, Okinawa, 903-0215 Japan; 3 Department of Otorhinolaryngology, Head and Neck Surgery, University of Miyazaki, Miyazaki, 889-1692 Japan

**Keywords:** Human papillomavirus, Oropharyngeal cancer, mTOR, Raptor, Rictor, Overall survival, Temsirolimus, Rapalog

## Abstract

**Background:**

Despite reports of a link between human papillomavirus (HPV) infection and mechanistic target of rapamycin (mTOR) signaling activation, the role of the mTOR pathway, especially raptor and rictor, in HPV-related head and neck cancer is still unclear. The aim of the present study was to elucidate the role of the mTOR pathway in HPV-related oropharyngeal squamous cell carcinoma (OPSCC).

**Methods:**

The present study involved two strategies. The first was to investigate the activity of mTOR and mTOR-related complexes in high-risk HPV-positive (UM-SCC47 and CaSki) and HPV-negative (SCC-4 and SAS) cancer cell lines. The second was to elucidate mTOR complex expression in 80 oropharyngeal cancer tissues and to examine the relationship between mTOR complex expression and survival in patients with OPSCC.

**Results:**

The UM-SCC47 and CaSki cell lines showed high gene and protein expression of raptor. They also exhibited G1/S and G2/M phase cell cycle arrest following 24 h incubation with 6 μM temsirolimus, a rapamycin analog, and temsirolimus administration inhibited their growth. HPV-related OPSCC samples showed high gene and protein expression of raptor and rictor compared with HPV-unrelated OPSCC. In addition, HPV-related OPSCC patients with high raptor and rictor expression tended to have a worse prognosis than those with low or medium expression.

**Conclusions:**

These results suggest that raptor and rictor have important roles in HPV-related OPSCC and that temsirolimus is a potential therapeutic agent for patients with HPV-related OPSCC. This is the first report to reveal the overexpression of raptor and rictor in HPV-related OPSCC.

**Supplementary Information:**

The online version contains supplementary material available at 10.1186/s12885-021-07794-9.

## Background

**Fig. 1:**
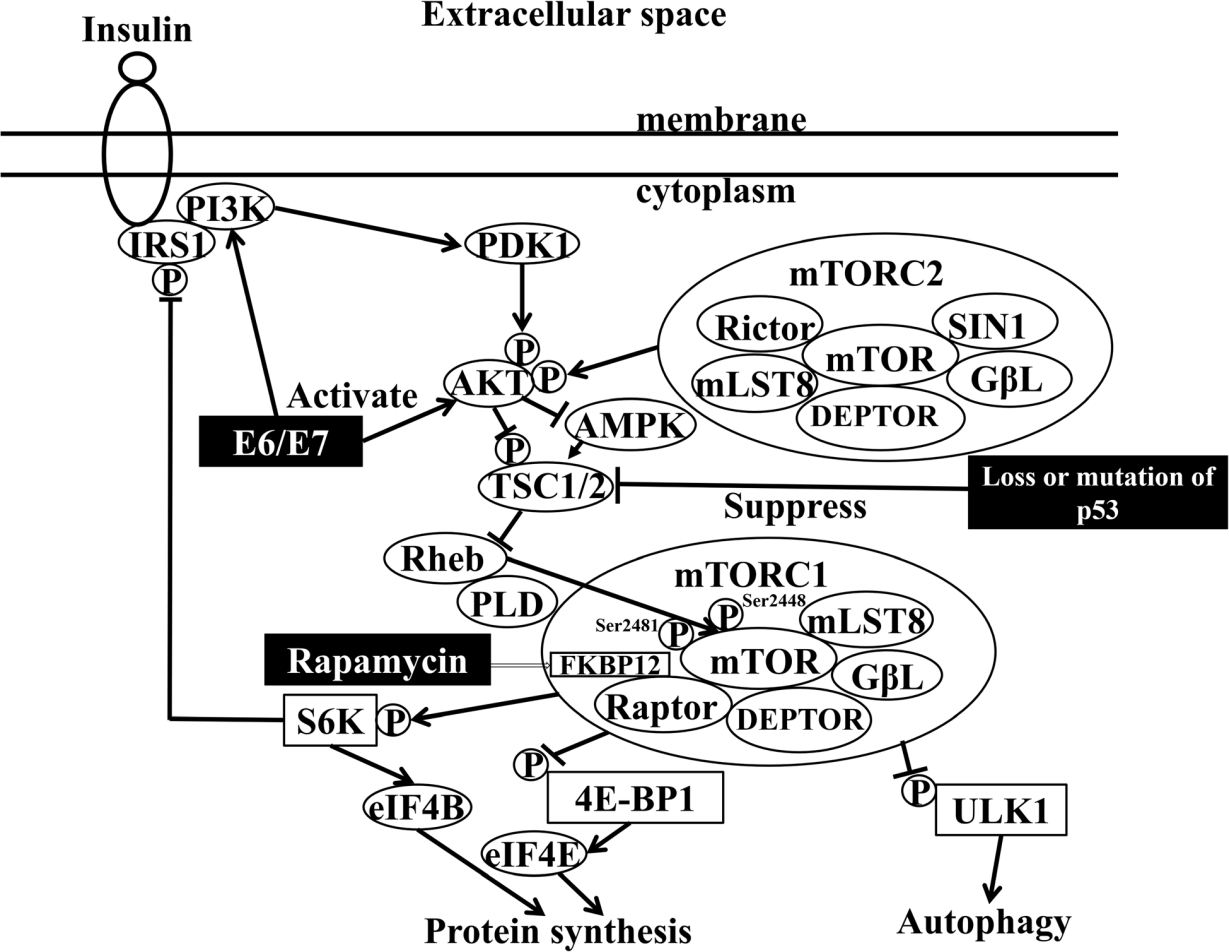
Schema of mTOR complexes 1 and 2 and their related molecules. AMPK, AMP-activated protein kinase; E6/E7, human papillomavirus E6 and E7; eIF4E, eukaryotic translation initiation factor 4E; ERK, extracellular signal-regulated kinase; FKBP12, 12-kDa FK506-binding protein; GβL, G-protein β-subunit like protein; GRB, growth factor receptor-bound protein; IRS1, insulin receptor substrate 1; MEK, mitogen-activated protein kinase/ERK kinase; mLST8, mammalian lethal with SEC13 protein 8; mTOR, mechanistic target of rapamycin; PDK1, 3-phosphoinositide-dependent protein kinase 1; PI3K, phosphatidylinositol 3-kinase; p, phosphorylation; p, dephosphorylation; PLD, phospholipase D; RAS, rat sarcoma; Rheb, Ras homolog enriched in brain; SIN1, stress-activated map kinase-interacting protein 1; SOS, son of sevenless homolog; S6, substrate ribosomal protein S6; TSC1/2, tuberous sclerosis 1/2; ULK1, unc-51-like kinase 1; 4E-BP1, eukaryotic translation initiation factor 4E binding protein 1

The mechanistic target of rapamycin (mTOR) forms two complexes, namely, mTOR complex 1 (mTORC1) and mTOR complex 2 (mTORC2) [1, 2]. mTORC1 has many well-established biological actions such as controlling protein and lipid synthesis, autophagy, energy metabolism, and cell survival/metabolism [3, 4]. In contrast, the functions of mTORC2 have not been fully clarified [5–7]. Proteins located downstream of mTORC1, such as eukaryotic translation initiation factor 4E binding protein 1 (4E-BP1) and eukaryotic translation initiation factor 4E (eIF4E) (Fig. 1), exert significant control over cap-dependent translation, cell growth, and cancer initiation and progression [8, 9]. Raptor is a scaffold protein that regulates the assembly, localization, and substrate binding (e.g., 4E-BP1 and p70 S6 kinase) of the mTORC1 complex [1, 9].

Inhibition of the mTOR signaling pathway reduces the proliferation and anchorage-independent growth of cancer cells and glioma cells [6, 10]. Rapamycin, which is an mTOR inhibitor, binds to 12-kDa FK506-binding protein (FKBP12), subsequently causing mTORC1 to dissociate from raptor, a component of mTORC1 [4]. In contrast, potent and selective ATP-competitive inhibitors of mTOR kinase, such as Torin 1 or 2, block the phosphorylation of mTORC1 and mTORC2. However, because these inhibitors are not specific to mTORC1 and mTORC2 [11], it is better to use rapamycin to investigate the different roles of mTORC1 and mTORC2 in cell lines. Rapamycin analogs (rapalogs) have been developed for the treatment of cancer; the first rapalog was temsirolimus, which was approved for the treatment of renal cell carcinoma by the US Food and Drug Administration in 2007 [12].

Head and neck cancer is the sixth most common cancer worldwide, accounting for approximately 5% of all cancer cases [13]. Excessive alcohol consumption and smoking are well-known risk factors for head and neck cancer [14, 15]. Although alcohol and tobacco use has decreased in recent years, the incidence of oropharyngeal cancer has nevertheless continued to increase [16]. The main cause of this phenomenon is the increase in the incidence of human papillomavirus (HPV)-related oropharyngeal cancer arising in the tonsillar region and base of the tongue [17]. HPV-related oropharyngeal squamous cell carcinoma (OPSCC) has a significantly better survival rate compared with other forms of OPSCC [15, 18, 19]. The clinical staging system for OPSCC separates HPV-related cancer from HPV-unrelated cancer in the 8th edition of the American Joint Committee on Cancer (AJCC) TNM classification [19, 20]. Because HPV-related OPSCC has a fair response to treatment, de-escalation treatment studies in HPV-related OPSCC are in progress [21–23].

Of the many signaling pathways related to tumor growth, the phosphoinositide 3-kinase (PI3K)/Akt/mTOR signaling pathway is frequently activated in cervical cancer and HPV-related head and neck squamous cell carcinoma (HNSCC) [24–31]. The administration of rapamycin alone significantly prolonged survival in an HPV-positive HNSCC murine model [29]. A single-arm multicenter phase II study of temsirolimus in platinum- and cetuximab-refractory recurrent and/or metastatic HNSCC showed that temsirolimus is a potential novel treatment paradigm for head and neck cancer [32]. However, HPV status was not predictive of success with temsirolimus treatment in that study. Despite reports of a link between HPV infection and mTOR signaling activation [24–30], the role of the mTOR pathway, especially raptor and rictor, which are key molecules in the mTOR pathway, in HPV-related HNSCC remains unclear. Therefore, the aim of this study was to elucidate the role of the mTOR pathway in HPV-related OPSCC.

## Methods

### Study design

The present study involved two strategies. The first was to investigate the activity of mTOR and mTOR-related complexes in high-risk (HR)-HPV-positive and -negative cancer cell lines. The second was to examine the expression of mTOR complexes in tissue from patients with OPSCC.

#### Cell lines and cell culture

**Fig. 2:**
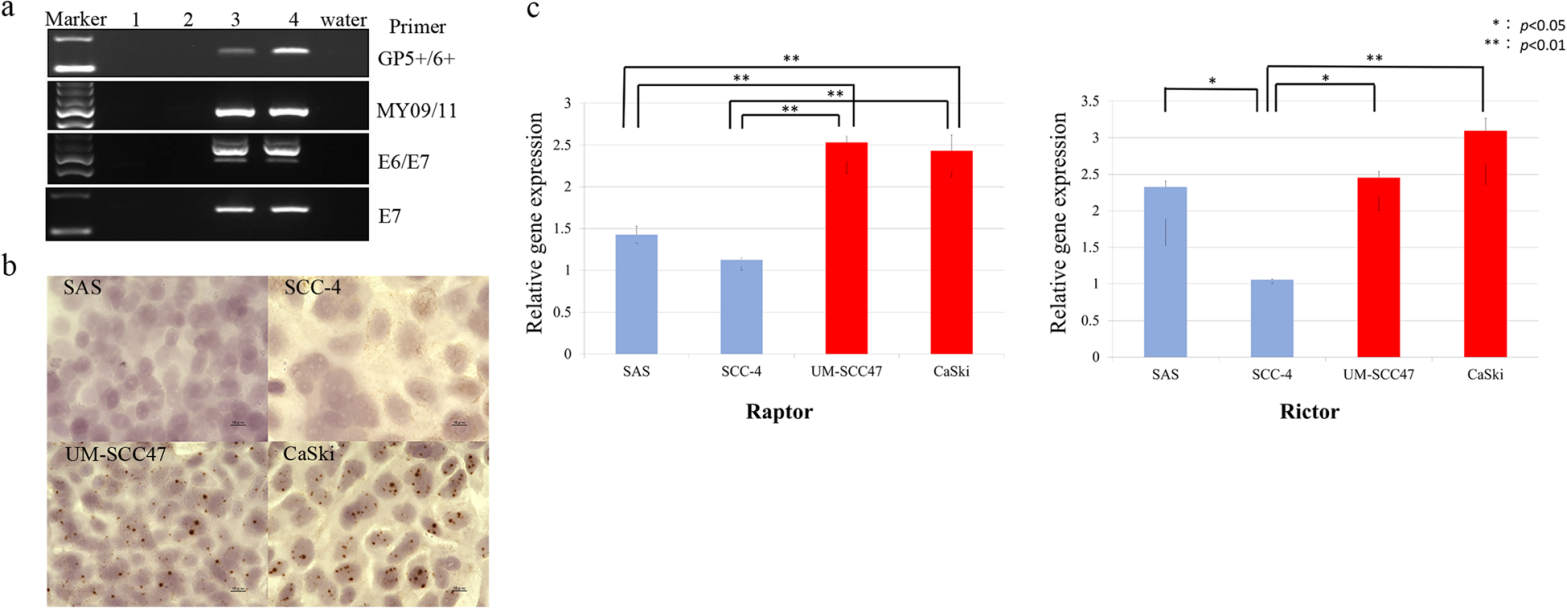
Cancer cell lines. **a** Analysis of HPV gene expression using primers GP5+/6+, MY09/11, HPV-16 E6/E7, and HPV-16 E7 (Table 1) in the cell lines employed in this study. Lane 1, SCC-4; Lane 2, SAS; Lane 3, UM-SCC47; Lane 4, CaSki. PCR amplicons were observed in the UM-SCC47 and CaSki cell lines. **b** In situ hybridization of HPV-16 DNA in the UM-SCC47 and CaSki cell lines. Brown dots inside cell nuclei were observed in both cell lines. The method was previously described in detail [31]. **c** Gene expression of raptor and rictor in the cell lines. Raptor was expressed at a higher level in the UM-SCC47 and CaSki cell lines than in SCC-4 and SAS cells. In the case of rictor, SCC-4 cells showed lower expression compared with the SAS, UM-SCC47, and CaSki cell lines. *p < 0.05; **p < 0.01. Cropped gels were used for conciseness of presentation. The original, uncropped gels are provided in Additional file 1

Human tongue carcinoma SAS and SCC-4 cell lines were used as HR-HPV-negative cell lines, whereas human oral cavity carcinoma UM-SCC47 and human cervical carcinoma CaSki cell lines were used as HR-HPV-positive cell lines. SAS and SCC-4 cells were obtained from the National Institute of Biomedical Innovation (JCRB Cell Bank, Osaka, Japan). CaSki cells were obtained from the European Collection of Authenticated Cell Cultures (Salisbury, UK). UM-SCC47 cells were isolated from a patient with lateral tongue cancer (a gift from Professor Thomas E. Carey, University of Michigan). The presence of HR-HPV DNA in the UM-SCC47 and CaSki cell lines was confirmed by PCR using the primers shown in Table 1 (Fig. 2a) and in situ hybridization of HR-HPV DNA (Fig. 2b) [33].

**Table 1:** Primers used in the present study

**Cloning primers**	**Sequence (5′–3′)**
GP5+	TTTGTTACTGTGGTAGATACTAC
GP6+	GAAAAATAAACTGTAAATCATATTC
MY09	CGTCCMARRGGAWACTGATC
MY11	GCMCAGGGWCATAAYAATGG
E6-F	AATGTTTCAGGACCCACAGG
E7-F	TGAAATAGATGGTCCAGCTGG
E7-R	TGCCCATTAACAGGTCTTCC
GAPDH-F	AATGGAAATCCCATCACC
GAPDH-R	CAGCCTTGGCAGCGCCAG
**Real-time PCR primers**	**Sequence (5′–3′)**
Raptor-F	ACTGATGGAGTCCGAAATGC
Raptor-R	TCATCCGATCCTTCATCCTC
Rictor-F	TGTATGCAAGAGCCAAGCAC
Rictor-R	CTGATTCCTGCTTTCCACAAG
β-Actin-F	CTCTTCCAGCCTTCCTTCCT
β-Actin-R	AGCACTGTGTTGGCGTACAG

SAS and SCC-4 cells were cultured in Dulbecco’s modified Eagle’s medium/F12 containing 10% fetal bovine serum, 2% 100 IU/mL penicillin, and 100 μg/mL streptomycin. UM-SCC47 and CaSki cells were cultured in RPMI-1640 medium containing 10% fetal bovine serum, 2% 100 IU/mL penicillin, and 100 μg/mL streptomycin. All cells were cultured at 37 °C in an incubator with 5% CO_2_.

#### Real-time PCR

The SAS, SCC-4, UM-SCC47, and CaSki cell lines were cultured in 6-well plates for 2 days at a density of 1.0 × 10^5^ cells/well with 2 mL medium. Total RNA was extracted from the cells using Isogen (Nippon Gene, Tokyo, Japan). A PrimeScript RT Reagent Kit with gDNA Eraser (Takara Bio, Shiga, Japan) was used to remove genomic DNA contamination and to synthesize cDNA from 500 ng total RNA. Real-time PCR was performed using the CFX-96 Real-Time PCR System (Bio-Rad, Hercules, CA). The relative mRNA expression of raptor and rictor was calculated with the ΔΔCt method using β-actin for normalization. A PCR reaction mixture (10 μL) contained 2 μL sample cDNA (10 ng/μL), 0.3 μM each of forward and reverse primers (Table 1), and 5 μL SYBR Premix Ex Taq II (Tli RNase H plus; Takara, Otsu, Japan). Amplification was carried out at 95 °C for 30 s, followed by 40 cycles at 95 °C for 5 s and 60 °C for 30 s. Specific amplification was verified by melting curve analysis and gel electrophoresis of the PCR products. All samples were measured in duplicate. For the ΔΔCt method, calibration curves for each gene were generated by using CaSki cDNA as a template. The PCR efficiency of raptor, rictor, and β*-*actin was 91.2, 117, and 93.4%, respectively (data not shown).

#### Western blot analysis

The SAS, SCC-4, UM-SCC47, and CaSki cell lines were cultured for 3 days at a density of 5.0 × 10^5^ cells per 25 cm^2^ with 5 mL medium, and the cells were 80% confluent after 3 days.

To evaluate PI3K/mTOR pathway activity under mTOR inhibition, each cell line was cultured at a density of 1.0 × 10^6^ cells in 10-cm dishes for 24 h. The medium was replaced with medium that did or did not contain 6 μM temsirolimus, and the cell lines were cultured for 24 h. Each cell line was pelleted and subjected to western bot analysis.

The cells were lysed in a buffer containing 62.5 mM Tris-HCl (pH 6.8), 2% sodium dodecyl sulfate (SDS), 10% glycerol, and 6% of 2-mercaptoethanol. Total protein concentration was determined using a DC Protein Assay Kit (Bio-Rad) with bovine serum albumin as the standard. Total protein (10 μg; 1 μg/μL) was separated by 10% SDS-polyacrylamide gel electrophoresis and transferred to polyvinylidene difluoride (PVDF) membranes (Bio-Rad). The membranes were blocked in PVDF Blocking Reagent for Can Get Signal® (TOYOBO Co., Ltd., Osaka, Japan) for 15 min and incubated with 1:1000 diluted primary antibodies (phospho-mTOR [Ser2448], phospho-mTOR [Ser2481], mTOR, raptor, rictor, G-protein β-subunit-like [GβL], phospho-Akt [Ser473], Akt, phospho-p70 S6 kinase [Thr389], p70 S6 kinase, phospho-tuberin/TSC2 [Thr1462], tuberin/TSC2, and phospho-4E-BP1 [Thr37/46], purchased from Cell Signaling Technology [Danvers, MA]; and phospho-4E-BP1 [Ser65], purchased from Santa Cruz Biotechnology [Dallas, TX]) at 4 °C overnight. Pan-actin expression (Cell Signaling Technology) was used as an internal control. The membranes were incubated with a secondary antibody (Cell Signaling Technology) for 1 h at room temperature. Finally, protein bands were detected using Clarity™ Western ECL Substrate (Bio-Rad) and processed by Image Lab software (Bio-Rad).

#### WST-1 assay

Each cancer cell line was counted using a Countess® Automated Cell Counter (Thermo Fisher Scientific, Waltham, MA) and grown at 4.0 × 10^6^ cells/mL in 96-well tissue culture plates in a total volume of 100 μL medium. After 24 h, the medium was replaced with medium containing 0–50 μM temsirolimus. At 24 h after medium change, 10 μL WST-1 reagent (Sigma-Aldrich, St. Louis, MO) was added to each well and incubated (37 °C, 0.5% CO_2_) for 1 h. The plate was read immediately at 440 nm with a reference reading at 600 nm (SH-1000 Lab Microplate Reader; Corona Electric, Ibaraki, Japan).

#### Flow cytometry

Each cancer cell line was counted using a Countess® Automated Cell Counter (Thermo Fisher Scientific) and grown at 2.5 × 10^5^ cells/mL in 6-well tissue culture plates in a volume of 2 mL medium. After 24 h, the medium was replaced with medium with or without (as a control) temsirolimus (6 μM). The cells were collected at 24 h after medium change. Flow cytometry was performed in accordance with the protocol of the BD Cycletest Plus DNA Kit (Becton Dickson, Franklin Lakes, NJ).

### Clinical tissue samples and clinical data

Real-time PCR was employed to measure raptor and rictor gene expression in clinical tissue samples from patients with OPSCC. A total of 98 treatment-naive OPSCC patients who underwent surgery or biopsy of a primary lesion from 2007 to 2016 were enrolled and analyzed retrospectively at the Department of Otorhinolaryngology, University of the Ryukyus Hospital, Japan. We followed the principles outlined in the Declaration of Helsinki for all human experimental investigations. Ethical approval was obtained from the University of the Ryukyus Research Ethics Committee (no. 2020–233), and informed consent was obtained from each patient.

All tissue samples from primary lesions were analyzed with PCR using fresh frozen samples and p16 immunohistochemistry using formalin-fixed paraffin-embedded samples. The detection methods have been previously described in detail [14, 16]. In the present study, the cutoff point for p16 overexpression was diffuse (≥75%) tumor expression, with at least moderate (+ 2/3) staining intensity, according to the 8th edition of the AJCC classification. The OPSCC patients were divided into three groups: HPV-related OPSCC (positive for p16 and HPV DNA expression), HPV-unrelated OPSCC (negative for p16 and HPV DNA expression), and others (positive for p16 or HPV DNA expression). The OPSCC patients were classified according to the 7th edition of the AJCC classification.

Total RNA was extracted from fresh frozen OPSCC samples using Isogen (Nippon Gene). A PrimeScript RT Reagent Kit with gDNA Eraser (Takara Bio) was used to remove genomic DNA contamination and to synthesize cDNA from 500 ng total RNA. Real-time PCR was performed using the CFX-96 Real-Time PCR System (Bio-Rad). The relative mRNA expression of raptor and rictor was calculated using the ΔΔCt method with β-actin for normalization. The PCR reaction mixture and amplification process were the same as described for the real-time PCR protocol used for the cell lines. All samples were measured in duplicate.

Raptor and rictor protein expression in clinical samples was evaluated by immunohistochemistry. Sections (4-μm thick) from paraffin-embedded block samples were deparaffinized in xylene and hydrated in a graded series of alcohol. Epitope retrieval was achieved by heating at 100 °C for 10 min in 1 mM EDTA buffer (pH 8.0). Endogenous peroxidase activity was quenched by incubating the sections in 0.3% H_2_O_2_ in methanol for 20 min at room temperature. A SAB-PO Kit (Nichirei Bioscience, Inc., Tokyo, Japan) was used to detect immunoreactivity to raptor and rictor according to the manufacturer’s protocol. After blocking non-specific reactions by incubation in 10% goat serum, the slides were incubated with a primary rabbit polyclonal anti-rictor antibody (1:500 dilution; #A300-459A; Bethyl Laboratories, Montgomery, TX) or rabbit polyclonal anti-raptor antibody (1:100 dilution; #PA5–85717; Thermo Fisher Scientific) overnight at 4 °C. Subsequently, a biotin-labeled secondary antibody and peroxidase-labeled streptavidin were applied. Immunolabeling was visualized by incubation in 3–3′-diaminobenzidine, and stained slides were counterstained with hematoxylin.

### Statistical analysis

Statistical analysis was carried out with SPSS 25.0 (SPSS, Inc., Chicago, IL). *P*-values less than 0.05 were considered significant. The results of the WST-1 assay, gene expression, and flow cytometry were analyzed using a χ^2^-test or Fisher’s exact test, as appropriate. Statistical analyses were made using Student’s *t*-test when homogeneity of variance of the variables was met between the two datasets, and using Welch’s *t*-test when homogeneity of variance was not met. Pearson’s χ^2^-test was used for categorical data, and the Mann–Whitney *U-*test was used for continuous variables. Cumulative survival was estimated using the Kaplan–Meier method and compared within the groups using the log-rank test.

## Results

### Cell line study

#### Real-time PCR

Real-time PCR was performed for quantitative analysis of raptor and rictor gene expression in the HR-HPV-negative and -positive cell lines (Fig. 2c). Raptor gene expression was higher in the HR-HPV-positive cell lines than in the HR-HPV-negative cell lines (SAS vs. UM-SCC47, *p* = 0.001; SAS vs. CaSki, *p* = 0.001; SCC-4 vs. UM-SCC47, *p* < 0.001; SCC-4 vs. CaSki, *p* < 0.001). Rictor gene expression was significantly lower in the SCC-4 cell line than in SAS, UM-SCC47, and CaSki cells. However, there was no significant difference in rictor expression between SAS and UM-SCC47 cells and between SAS and CaSki cells. The complete PCR results for HPV are provided as Additional file 1.

#### Western blot analysis

**Fig. 3:**
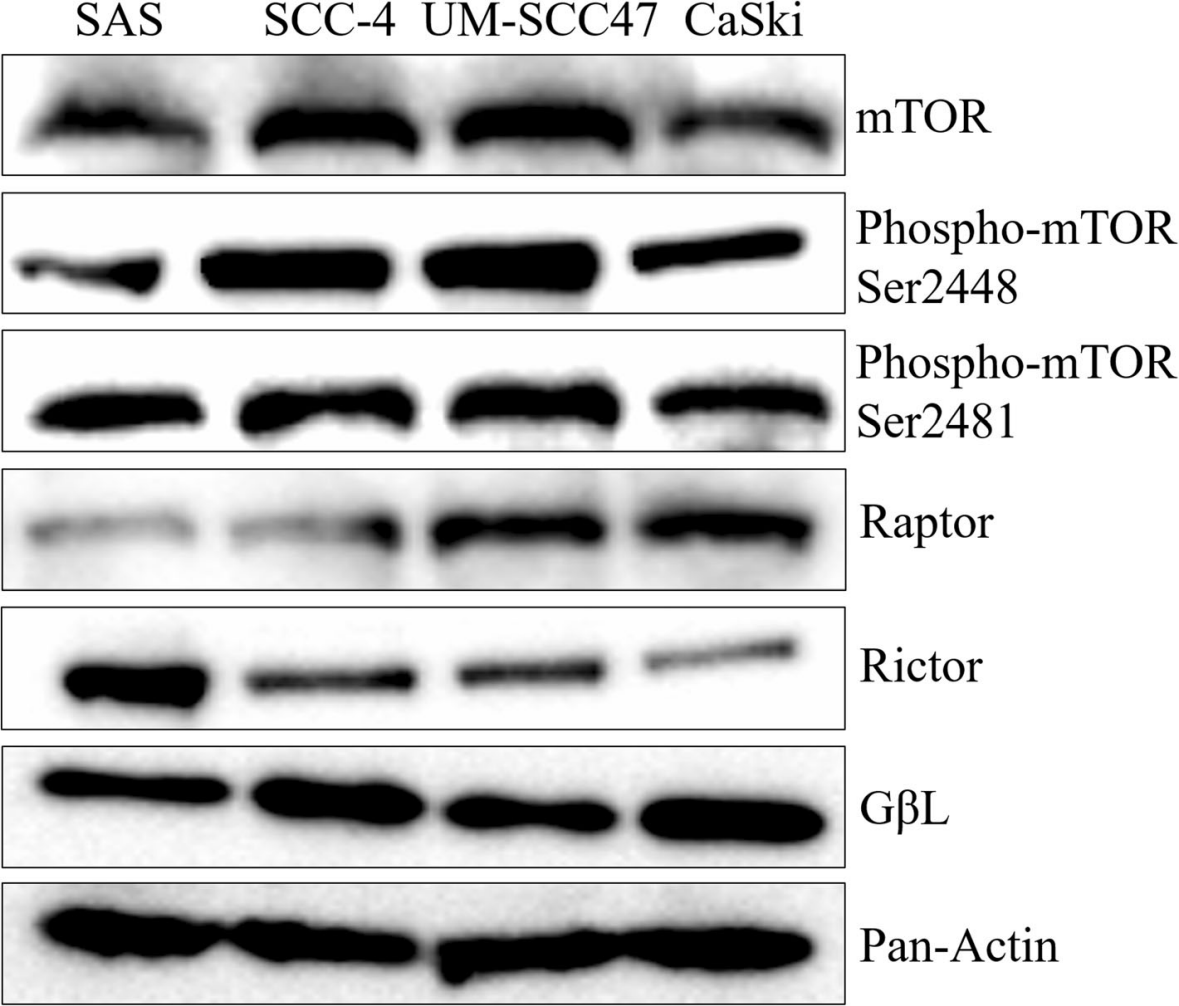
mTOR-related protein production detected by western blotting in the cell lines. High raptor expression was observed in the UM-SCC47 and CaSki cell lines, whereas high rictor expression was observed in the SAS cell line. Cropped blots were used for conciseness of presentation. The original, uncropped blots are provided in Additional file 2

Protein expression of mTOR components was investigated in the HR-HPV-negative (SAS and SCC-4) and HR-HPV-positive (UM-SCC47 and CaSki) cell lines (Fig. 3). The internal control pan-actin was detected equally in all cell lines. There were no differences in the expression of mTOR, phospho-mTOR [Ser2448], phospho-mTOR [Ser2481], and GβL between the HR-HPV-positive and -negative cell lines. The HR-HPV-positive cell lines UM-SCC47 and CaSki showed higher raptor expression compared with the SAS and SCC-4 cell lines. On the contrary, the HR-HPV-negative cell line SAS showed higher rictor expression than the SCC-4, UM-SCC47, and CaSki cell lines.

**Fig. 4:**
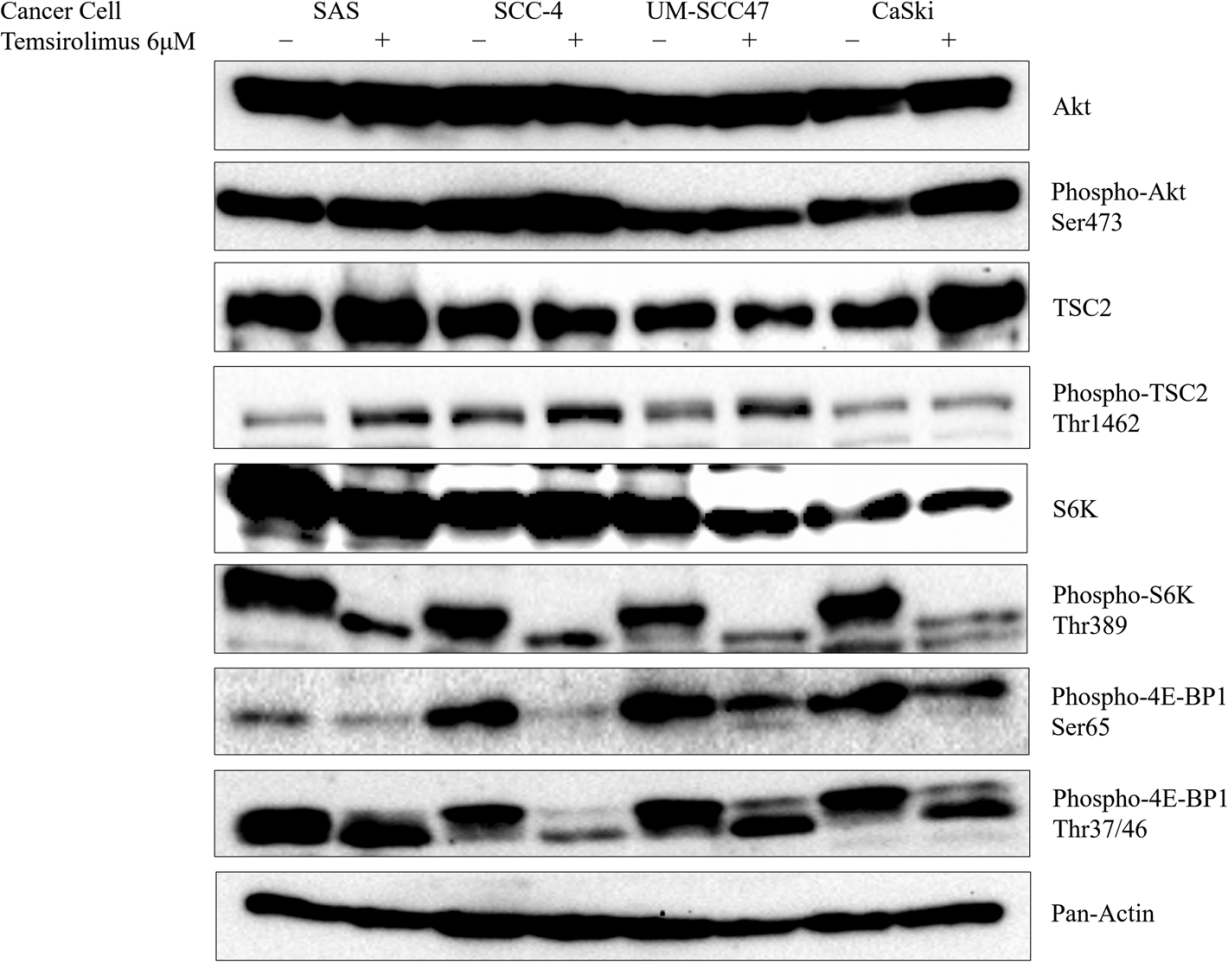
Change in the levels of downstream and upstream proteins of the mTOR pathway under temsirolimus treatment in four cancer cell lines. There was no difference in the levels of Akt, phospho-Akt [Ser473], and pan-actin among the four cell lines. Phospho-p70 S6 kinase production was lowered by temsirolimus treatment in each cell line. Temsirolimus treatment increased phospho-TSC2 [Thr462] and decreased phospho-4E-BP1 [Ser65 and Thr37/46] levels in the HR-HPV-positive UM-SCC47 cell line. The original, uncropped blots are provided in Additional file 2

Expression of proteins located downstream and upstream of mTOR (Fig. 1) was investigated in the presence and absence of 6 μM temsirolimus (Fig. 4). Expression of phospho-p70 S6 kinase [Thr389] was decreased by temsirolimus treatment in all four cell lines. Although the levels of phospho-4E-BP1 [Ser65] and phospho-4E-BP1 [Thr37/46] were decreased by temsirolimus treatment of the UM-SCC47, CaSki, and SCC-4 cell lines, there was no obvious change in their levels in the SAS cell line.

Expression of phospho-Akt [Ser473], which is located upstream of mTORC1, was increased slightly by temsirolimus treatment, except in the UM-SCC47 cell line. The levels of phospho-TSC2 [Thr1462], which is also located upstream of mTORC1, were increased by temsirolimus treatment in the UM-SCC47, SAS, and SCC-4 cell lines. However, there was no apparent change in phospho-TSC2 levels under temsirolimus treatment in the CaSki cell line.

#### WST-1 assay

**Fig. 5:**
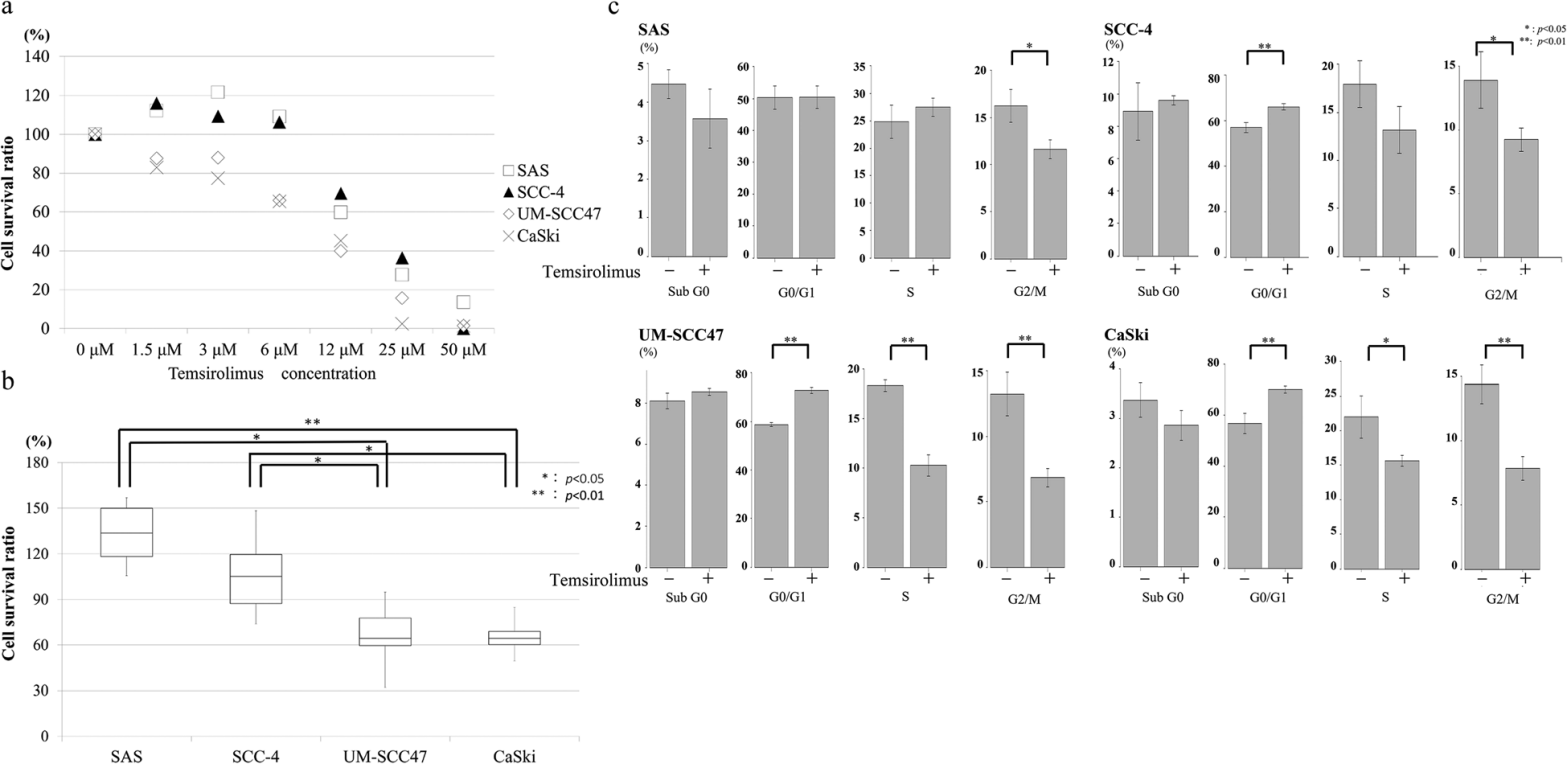
Effects of incubation with temsirolimus. **a** Cell survival ratios in the WST-1 assay after 24-h incubation with various concentrations of temsirolimus. Although the cell survival ratios of each cell line were slightly different, ≥12 μM temsirolimus inhibited cell survival in all cells. A prominent difference in cell survival among the cell lines was observed at 6 μM temsirolimus. **b** Box plots of cell survival rates in the cell lines after incubation with 6 μM temsirolimus for 24 h. The cell survival ratios of the UM-SCC47 and CaSki cell lines were significantly worse than those of the SCC-4 and SAS cell lines. *p < 0.05; **p < 0.01. **c** Temsirolimus induces cell cycle arrest in the UM-SCC47 and CaSki cell lines. UM-SCC47, CaSki, and SCC-4 cells were arrested at the G0/G1 and G2/M phases by incubation with temsirolimus and the number of cells in the S phase was significantly reduced in the UM-SCC47 and CaSki cell lines. In contrast, only SAS cells showed a decrease in the number of cells in the G2/M phase. *p < 0.05; **p < 0.01. The original flow cytometry data are provided in Additional file 3

The relationship between the cell survival rate and temsirolimus concentration after 24-h incubation is shown in Fig. 5a. Concentrations of temsirolimus ≥12 μM reduced the survival rate of all four cell lines. SCC-4 and SAS cells had almost identical cell survival curves under various temsirolimus concentrations; they showed a slight increase in survival at 3 μM temsirolimus, but survival decreased at > 12 μM temsirolimus. In contrast, the survival rate of UM-SCC47 and CaSki cells tended to decrease as the concentration of temsirolimus increased. The most prominent difference between the HR-HPV-positive and -negative cell lines in cell survival rates was observed at 6 μM temsirolimus. The cell survival rate was higher in SCC-4 and SAS cells than in UM-SCC47 and CaSki cells (SCC-4 vs. UM-SCC47, *p* = 0.016; SCC-4 vs. CaSki, *p* = 0.014; SAS vs. UM-SCC47, *p* = 0.010; SAS vs. CaSki, *p* = 0.009) (Fig. 5b).

#### Flow cytometry

Flow cytometry was used to determine the influence of temsirolimus on the cell cycle of the cell lines (Fig. 5c). There was a difference in the cell cycle distribution in the UM-SCC47, CaSki, and SCC-4 cell lines at stages GO/G1 (UM-SCC47, *p* < 0.001; CaSki, *p* = 0.005; SCC-4, *p* = 0.004), S (UM-SCC47, *p* < 0.001; CaSki, *p* = 0.025; SCC-4, *p* = 0.075), and G2/M (UM-SCC47, *p* = 0.003; CaSki, *p* = 0.003; SCC-4, *p* = 0.028) between the presence and absence of temsirolimus. Thus, there was a higher rate of G0/G1 and lower rates of S and G2/M in the UM-SCC47, CaSki, and SCC-4 cell lines in the presence of temsirolimus compared with its absence. Furthermore, there was a slight difference of cell cycle distribution in the SAS cell line between the presence and absence of temsirolimus at each stage of the cell cycle, except for G2/M (*p* = 0.017).

### Clinical sample study

#### Patient characteristics

There were 25 patients with HPV-related OPSCC (HPV DNA presence and p16 overexpression), 55 with HPV-unrelated OPSCC (neither HPV DNA nor p16 overexpression), and 18 with undetermined OPSCC (HPV DNA presence or p16 overexpression). There was no significant difference in age, sex, and subsites between the HPV-related and -unrelated patient groups (Table 2). The chemotherapy (platinum and 5-fluorouracil) and irradiation protocol used in this study was previously reported [15]. The HPV-unrelated group had a higher T-classification rate than the HPV-related group (*p* = 0.009) and was more likely to receive a surgical procedure as primary treatment (*p* = 0.012).

**Table 2:** Clinical characteristics of the patients with OPSCC

	All patients (*n* = 80)	HPV-related (*n* = 25)	HPV-unrelated (*n* = 55)	*P-*value
Sex				0.595
Male	61	20 (80.0)	41 (74.5)	
Female	19	5 (20.0)	14 (25.5)	
Age (years)				0.513
	63.2 ± 10.2	62.0 ± 8.9	63.7 ± 10.9	
T				0.009
T1, T2	36	16 (64.0)	18 (32.7)	
T3, T4	49	9 (36.0)	37 (67.3)	
N				0.14
N0, N1	33	7 (28.0)	25 (45.4)	
N2, N3	52	18 (72.0)	30 (54.6)	
M				0.459
M0	76	25 (100)	51 (92.7)	
M1	2	0 (0)	2 (7.3)	
SCC differentiation				0.103
Well	22	5 (20.0)	17 (30.9)	
Moderately	42	11 (44.0)	31 (56.4)	
Poorly	8	5 (20.0)	3 (5.4)	
Unknown	8	4 (16.0)	4 (7.3)	
Tumor subsite				0.134
Lateral	59	22 (88.0)	37 (67.3)	
Anterior	19	3 (12.0)	16 (29.1)	
Superior	2	0 (0)	2 (3.6)	
Primary treatment				0.012
Surgery ± RT/CCRT CCRT to Surgery	23	3 (12.0)	20 (36.4)	
RT or CCRT	52	22 (88.0)	30 (54.5)	
No treatment	5	0 (0)	5 (9.1)	
5-year overall survival (%)	73.7	95.8	63.2	0.024

*CCRT* Concurrent chemoradiotherapy, *RT* Radiotherapy, *SCC* Squamous cell cancer

#### Real-time PCR

**Fig. 6:**
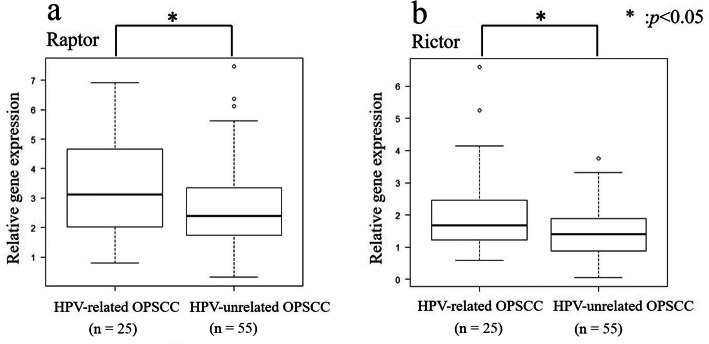
Relative mRNA expression of raptor and rictor in 25 HPV-related and 55 HPV-unrelated OPSCC samples. Samples obtained from HPV-related OPSCC patients showed significantly higher expression of raptor (**a**) and rictor (**b**). *p < 0.05

Gene expression was compared between the HPV-related and -unrelated OPSCC patients (Fig. 6). Raptor (*p* = 0.046) and rictor (*p* = 0.017) gene expression was higher in the HPV-related OPSCC group than in the HPV-unrelated OPSCC group.

#### Immunohistochemical analysis of raptor and rictor expression

**Fig. 7:**
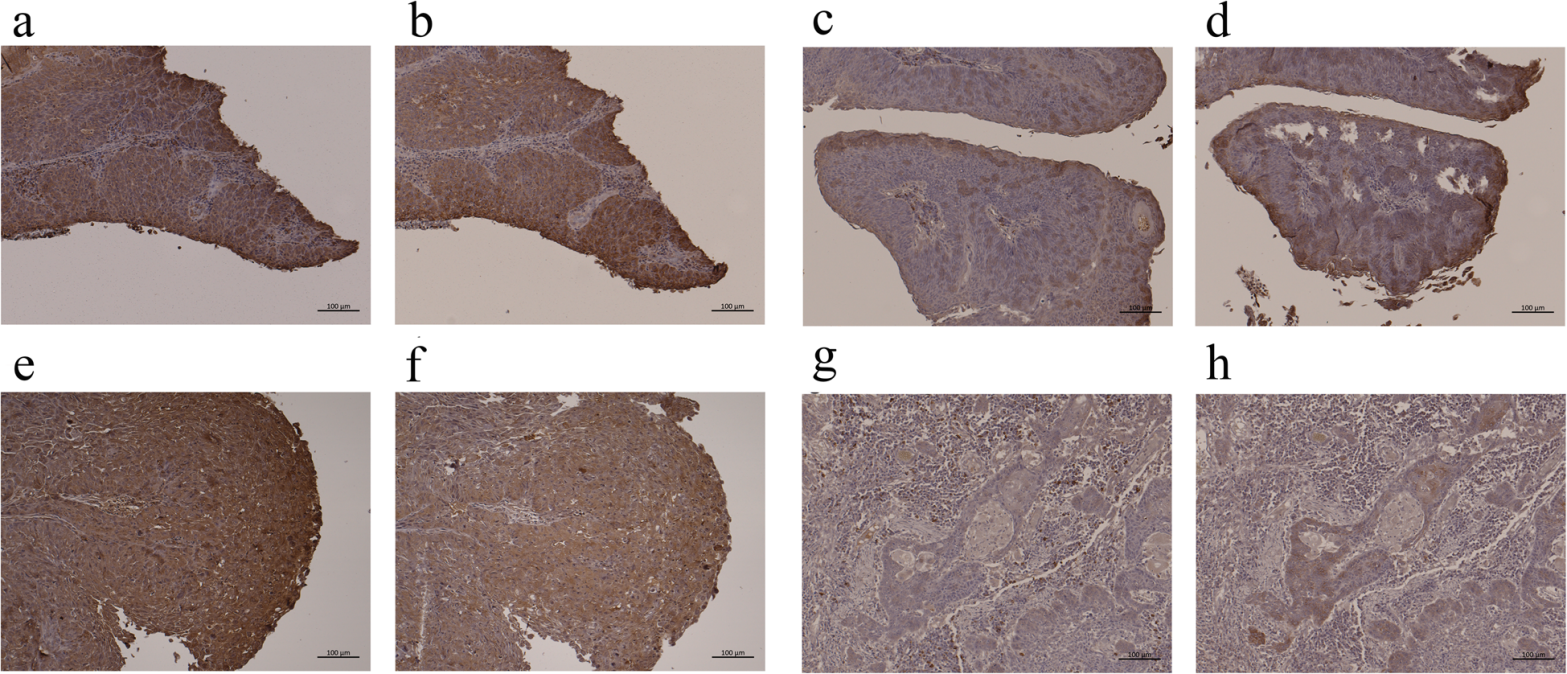
Immunohistochemical analysis of raptor and rictor expression. **a** Raptor expression in a representative case of HPV-related OPSCC with high raptor expression in real-time PCR (∆∆CT = 4.29). Strong raptor immunoreactivity was also observed in the cytosol of cancer cells. Bar, 100 μm. **b** Rictor expression in a representative case of HPV-related OPSCC with high rictor expression in real-time PCR (∆∆CT = 5.24). The sample was obtained from the same patient as in **a**. Strong rictor immunoreactivity was also observed in the cytosol of cancer cells. Bar, 100 μm. **c** Raptor expression in a representative case of HPV-related OPSCC with low raptor expression in real-time PCR (∆∆CT = 0.81). Weak raptor immunoreactivity was also observed in the cytosol of cancer cells. Bar, 100 μm. **d** Rictor expression in a representative case of HPV-related OPSCC with low rictor expression in real-time PCR (∆∆CT = 1.29). The sample was obtained from the same patient as in **c**. Weak rictor immunoreactivity was also observed in the cytosol of cancer cells. Bar, 100 μm. **e** Raptor expression in a representative case of HPV-unrelated OPSCC with high raptor expression in real-time PCR (∆∆CT = 3.37). Strong raptor immunoreactivity was also observed in the cytosol of cancer cells. Bar, 100 μm. **f** Rictor expression in a representative case of HPV-unrelated OPSCC with high rictor expression in real-time PCR (∆∆CT = 2.29). The sample was obtained from the same patient as in **e**. Strong rictor immunoreactivity was also observed in the cytosol of cancer cells. Bar, 100 μm. **g** Raptor expression in a representative case of HPV-unrelated OPSCC with low raptor expression in real-time PCR (∆∆CT = 1.03). Weak raptor immunoreactivity was also observed in the cytosol of cancer cells. Bar, 100 μm. **h** Rictor expression in a representative case of HPV-unrelated OPSCC with low rictor expression in real-time PCR (∆∆CT = 0.75). Weak rictor immunoreactivity was also observed in the cytosol of cancer cells. Bar, 100 μm

Immunohistochemical evaluations were performed in 5 patients who had high raptor and rictor gene expression and in 5 patients who had low raptor and rictor gene expression (Fig. 7).

Raptor and rictor expression were observed in the cytoplasm of cancer cells. The HPV-related OPSCC patients with high gene expression of raptor and rictor also demonstrated high protein expression in immunohistochemistry, whereas HPV-unrelated OPSCC patients with low gene expression showed low protein expression in immunohistochemistry.

#### Survival estimation in relation to raptor and rictor expression

According to raptor and rictor expression in real-time PCR, OPSCC patients were divided into high expression (>two-thirds), medium expression (equal to one-third to two-thirds), or low expression (≤one-third). For raptor expression, there were 6 HPV-related OPSCC patients with low expression, 7 with medium expression, and 12 with high expression, whereas 19 HPV-unrelated OPSCC patients had low expression, 20 had medium expression, and 16 had high expression. For rictor expression, there were 6 HPV-related OPSCC patients with low expression, 9 with medium expression, and 10 with high expression, whereas 21 HPV-unrelated OPSCC patients had low expression, 17 had medium expression, and 17 had high expression.

**Fig. 8:**
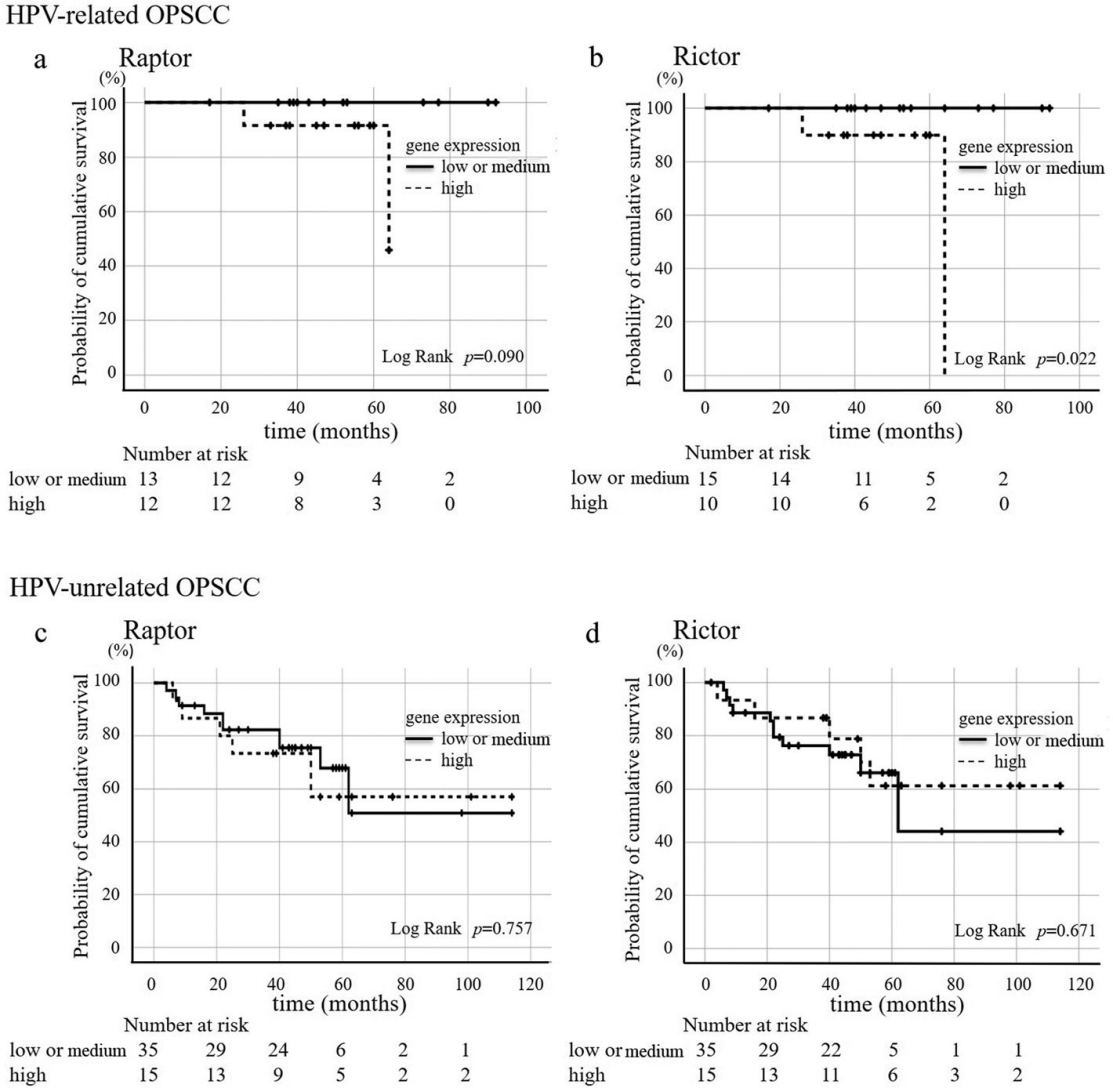
Kaplan-Meier curve of HPV-related and -unrelated OPSCC patients according to raptor and rictor expression. **a** Raptor expression and cumulative overall survival in HPV-related OPSCC patients. Patients with high raptor expression tended to show worse cumulative overall survival compared with those with low or medium expression, but the difference did not reach statistical significance. **b** Rictor expression and cumulative overall survival in HPV-related OPSCC patients. Patients with high rictor expression had significantly worse cumulative overall survival compared with those with low or medium expression. **c** Raptor expression and cumulative overall survival in HPV-unrelated OPSCC patients. There was no difference in cumulative overall survival in HPV-unrelated OPSCC patients according to raptor expression. Five cases were removed from the survival estimation because of poor general condition. **d** Rictor expression and cumulative overall survival in HPV-unrelated OPSCC. There was no difference in cumulative overall survival in HPV-unrelated OPSCC patients according to rictor expression. Five cases were removed from the survival estimation because of poor general condition

HPV-related and -unrelated OPSCC patients were divided into “high” and “medium or low” raptor or rictor expression groups for survival estimation (Fig. 8). Five patients who could not be treated were excluded from this estimation. Patients with HPV-related OPSCC (25 cases) had a better prognosis than those with HPV-unrelated OPSCC (50 cases), shown in Table 2 (*p* = 0.024). HPV-related OPSCC patients with high raptor expression (12 cases) tended to show lower cumulative overall survival (*p* = 0.090, Fig. 8a), whereas those with high rictor expression (10 cases) showed poor cumulative overall survival *(p* = 0.022, Fig. 8b). However, there was no close relationship between raptor or rictor expression and cumulative overall survival in patients with HPV-unrelated OPSCC (Fig. 8c, d).

## Discussion

This study demonstrated activation of the mTOR pathway in HPV-positive head and neck cancer cell lines as well as HPV-negative cell lines in vitro. mTORC1 and mTORC2 are known to show different sensitivities to rapamycin, and the efficacy of rapamycin varies in different cell lines according to dosage [11, 34]. One of the reasons for this is that nanomolar concentrations of rapamycin are sufficient for suppressing p70 S6 kinase phosphorylation, whereas the suppression of 4E-BP1 phosphorylation requires micromolar concentrations of rapamycin [11, 35]. In the present study, treatment with 6 μM of the rapalog temsirolimus successfully reduced the phosphorylation of p70 S6 kinase in all four cell lines and reduced the phosphorylation of 4E-BP1 in the UM-SCC47, CaSki, and SCC-4 cell lines.

The UM-SCC47, CaSki, and SCC-4 cell lines also showed a high proportion of cells at the G0/G1 phase of the cell cycle and a low proportion of cells at the S and G2/M phases after 24-h incubation with 6 μM temsirolimus, suggesting the induction of cell cycle arrest at G1/S and G2/M by temsirolimus treatment. Of the four cell lines tested, the HPV-positive head and neck cancer cell line UM-SCC47 had several unique characteristics, such as high raptor expression, and treatment with 6 μM temsirolimus inhibited p70 S6 kinase and 4E-BP1 phosphorylation and the activation of TSC2. The suppression of mTOR results in G1 cell cycle arrest. A key downstream target of AMPK is TSC2. AMPK phosphorylates TSC2 and activates its GAP activity [36], resulting in the suppression of Rheb due to the hydrolysis of bound GTP to GDP (Fig. 1). Rapamycin treatment reverses feedback suppression, inducing upstream receptor tyrosine kinase signaling, and activates Akt by the growth factor-dependent phosphorylation of Akt at the Ser473 mTORC2 site [37, 38]. However, we found that temsirolimus treatment did not affect Akt phosphorylation and induced the phosphorylation of TSC2 in UM-SCC47 cells.

Cancer progression has a close relationship with mTORC1/2 [2, 39]. Rapalogs such as temsirolimus inhibit the activity of mTOR when bound to FKBP12 by causing mTOR to dissociate from raptor [31]. A previous study in gastric cancer cells suggested that knockdown of raptor expression significantly reduces cell proliferation and induces G0/G1-phase cell cycle arrest [40]. Another study showed the efficacy of the mTOR inhibitor everolimus in postmenopausal hormone receptor-positive advanced breast cancer [41]. The authors used everolimus in combination with endocrine therapy and their results demonstrated improved progression-free survival compared with treatment without everolimus. A double-blind, randomized, placebo-controlled phase III trial with everolimus also showed prolonged progression-free survival relative to placebo in patients with metastatic renal cell carcinoma whose disease had progressed on vascular endothelial growth factor-targeted therapy [42]. Although there have been a number of reports describing mTOR activation in 80 to 90% of patients with head and neck cancer [7, 24, 27, 28, 43], little is known about its role in HPV-related OPSCC. Further study is needed to clarify the background of these phenomena observed in UM-SCC47 cells.

In accordance with our in vitro studies, clinical HPV-related OPSCC samples showed high raptor and rictor gene and protein expression compared with HPV-unrelated OPSCC samples in vivo. Raptor and rictor expression was immunohistologically evaluated based on mRNA expression detected by real-time PCR of raptor and rictor. In survival analysis, HPV-related OPSCC patients with high raptor and rictor expression tended to have a worse prognosis than those with low or middle expression. Raptor is a scaffold protein that regulates the assembly, localization, and substrate binding (e.g., 4E-BP1 and p70 S6 kinase) of the mTORC1 complex [1, 9, 44]. The raptor-mediated translocation of mTOR to lysosomes is an important step for the activation of mTORC1 [38, 45, 46]. A recent study reported that the acute or short-term treatment of several cell lines with rapamycin dissociates not only raptor but also rictor from mTOR, suggesting the inhibition of mTORC1 and mTORC2 [47]. Although no report has suggested raptor or rictor as prognostic markers in head and neck cancer, in hepatocellular carcinoma, high levels of mTOR and rictor mRNA are an indicator of early recurrence after hepatic resection [48]. Several studies on the use of rapalogs for treating head and neck cancer have reported that temsirolimus has therapeutic potential [32, 49]; however, these reports lacked information on the HPV infection status of their patients. This is the first report to reveal the overexpression of raptor and rictor and the survival outcome in relation to raptor and rictor expression in patients with HPV-related OPSCC. Because the number of patients in this study was limited, further study is needed to clarify the importance of raptor and rictor expression in head and neck cancers in relation to HPV infection.

The viral E6 and E7 proteins are important oncoproteins in HPV-related cancers. Since the mechanism of carcinogenesis is quite different between HPV-related and -unrelated OPSCC, they might have different target molecules for treatment, such as rapalogs (Fig. 1). Although HPV-related OPSCC demonstrates a fair 5-year overall survival rate, patients with HPV-related OPSCC are usually younger than those with HPV-unrelated OPSCC [50, 51]. There is interest in the development of additional molecular target therapies for HPV-related OPSCC in view of their reduced toxicity. In the present study, high raptor and rictor expression in HPV-related OPSCC tended to show a correlation with a poor prognosis, compared with low or medium expression. These results suggest that raptor has an important role in HPV-related OPSCC and that temsirolimus is a potential therapeutic agent for patients with HPV-related OPSCC. Knockdown of rictor expression inhibits mTORC2 activity and the proliferation of tumor cells and subsequent tumor growth in glioma cells [10]. In contrast, overexpression of rictor in glioma cell lines increases the assembly and activity of mTORC2 [6]. In the present study, although rictor mRNA expression was significantly higher in the SAS, UM-SCC47, and CaSki cell lines than in SCC-4 cells, rictor protein expression was lower in the UM-SCC47 and CaSki cell lines than in SAS cells. This discrepancy in rictor expression needs to be clarified in head and neck cancer cell lines to determine the underlying mechanism. In addition, further investigations are needed to evaluate the role of rictor in the cell cycle arrest observed following incubation with temsirolimus.

## Conclusions

The results of this study suggest that raptor and rictor have important roles in HPV-related OPSCC and that temsirolimus is a potential therapeutic agent for patients with HPV-related OPSCC. This is the first report to reveal the overexpression of raptor and rictor in HPV-related OPSCC.

## Supplementary Information

**Additional file 1.** PCR results for HPV detection in the cell lines.

**Additional file 2.** The original, uncropped blots shown in part in Figs. 3 and 4.

**Additional file 3.** The original flow cytometry data used in Fig. 5c. P1, sub G0 phase; P2, G0/G1 phase; P3, S phase; P4, G2/M phase; tem(−), no temsirolimus treatment; tem(+), temsirolimus treatment.

## Data Availability

Full-length gels and blots shown in Figs. 2, 3, and 4 are provided as Additional file 2. The datasets generated and/or analyzed during the current study shown in Table 2 have not been made publicly available at present because we have not been granted permission by the institutional review board to do so. However, data can be made available from the corresponding author upon reasonable request.

## Abbreviations

4E-BP1: Eukaryotic translation initiation factor 4E binding protein 1
AJCC: American Joint Committee on Cancer
eIF4E: Eukaryotic translation initiation factor 4E
FKBP12: 12-kDa FK506-binding protein
HNSCC: Head and neck squamous cell carcinoma
HR: High-risk
HPV: Human papillomavirus
mTOR: Mechanistic target of rapamycin
mTORC: mTOR complex
OPSCC: Oropharyngeal squamous cell carcinoma
PI3K: Phosphoinositide 3-kinase
PVDF: Polyvinylidene difluoride
rapalog: Rapamycin analog
