# Supplementary figures and images for: Raptor and rictor expression in patients with human papillomavirus-related oropharyngeal squamous cell carcinoma
Source: BMC Cancer. 2021 Jan 22;21:87. doi: 10.1186/s12885-021-07794-9 (PMC7821513; doi:10.1186/s12885-021-07794-9)

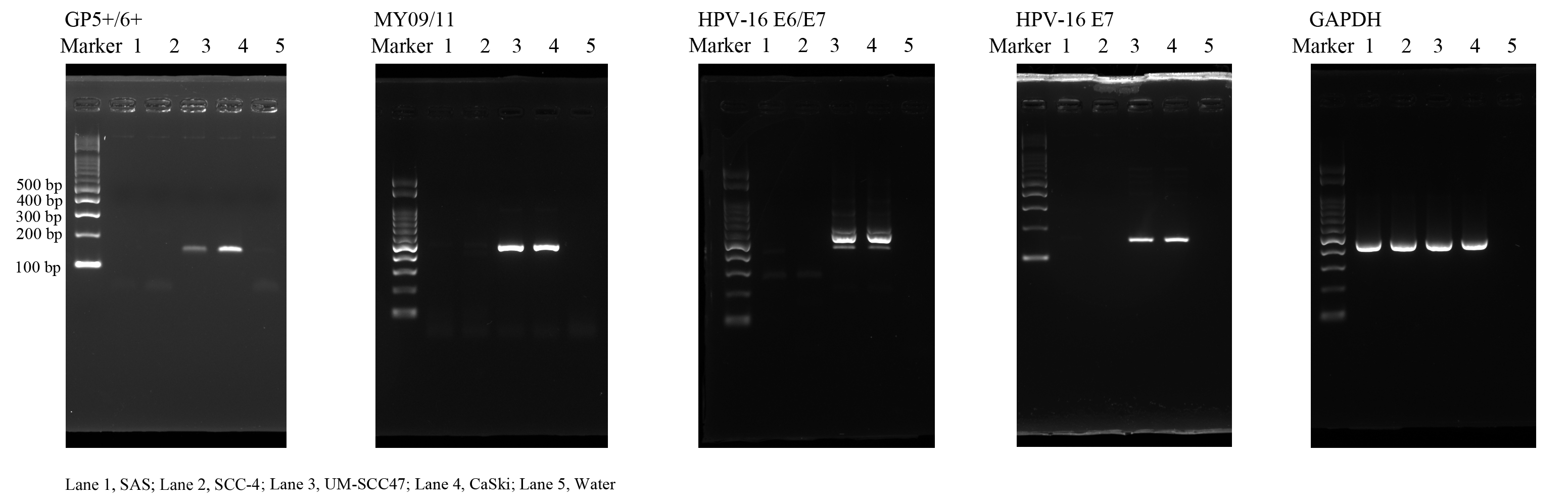

Supplement: Supplementary file 1 — Additional file 1. PCR results for HPV detection in the cell lines. [file 12885_2021_7794_MOESM1_ESM.tif]

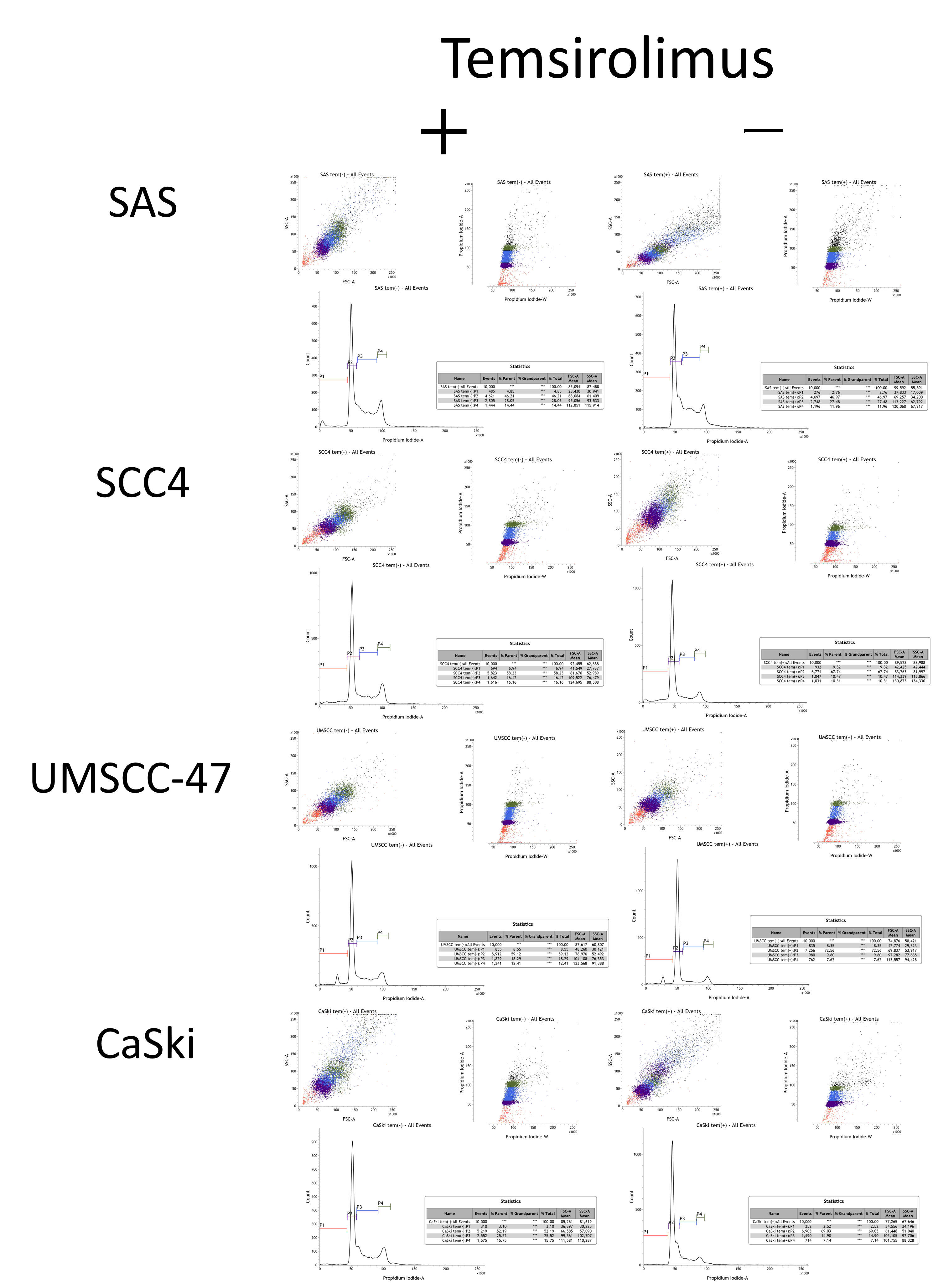

Supplement: Supplementary file 3 — Additional file 3. The original flow cytometry data used in Fig. 5c. P1, sub G0 phase; P2, G0/G1 phase; P3, S phase; P4, G2/M phase; tem(−), no temsirolimus treatment; tem(+), temsirolimus treatment. [file 12885_2021_7794_MOESM3_ESM.tif]
